# Identification of biomarkers for pediatric sepsis based on machine learning and bioinformatics analysis

**DOI:** 10.3389/fimmu.2026.1768323

**Published:** 2026-04-07

**Authors:** Weidong Ye, Sijia Chen, You Duan, Junwei Shan, Jian Wang, Heng Xu, Zhongyan Li, Cheng Guo

**Affiliations:** 1 Department of Pediatric, The Quzhou Affiliated Hospital of Wenzhou Medical University, Quzhou People’s Hospital, Quzhou, Zhejiang, China; 2 West China Institute of Women and Children’s Health, West China Second University Hospital, Sichuan University, Chengdu, China; 3 Huante Biotechnology Co. Ltd., Hangzhou, China; 4 Department of Laboratory Medicine, West China Hospital, Sichuan University, Chengdu, Sichuan, China

**Keywords:** bioinformatics analysis, biomarkers, machine learning, pediatric sepsis, RORA

## Abstract

Pediatric sepsis is a systemic inflammatory syndrome caused by dysregulated host immune responses, with a high mortality rate and a lack of effective biomarkers, posing significant challenges for early diagnosis and treatment. This study integrated six bulk RNA-seq datasets related to pediatric sepsis, including 497 patients and 116 healthy control samples. Weighted gene co-expression network analysis was used to identify gene modules significantly associated with pediatric sepsis, and 237 high-confidence biomarkers were screened based on 14 machine learning models, among which RORA and GPR183 stood out in multiple models. Functional analysis indicated that these biomarkers were mainly involved in biological processes such as transcription and translation, the immune system, and cellular senescence. Immune infiltration analysis revealed a significant reduction in adaptive immune cells such as B cells and CD8^+^ T cells and an increase in neutrophil and monocyte infiltration in pediatric sepsis patients, consistent with the “immunoparalysis” theory. Notably, RORA and GPR183 were positively correlated with CD8^+^ T cells, suggesting their potential role in regulating T cell function. Additionally, we developed an open-source website for real-time application of biomarkers. Furthermore, we established a sepsis model in zebrafish and found that *rora1*, *rora2*, and *gpr183* expression levels were significantly downregulated in the disease group. This study provides new insights for developing novel diagnostic tools and targeted therapies for pediatric sepsis.

## 1 Introduction

Sepsis, a systemic inflammatory response syndrome caused by dysregulation of the host’s response to infection, represents one of the major challenges in global child health (1). Its high mortality and long-term disability rates make it a leading cause of childhood death, particularly in resource-limited settings (2). Early recognition and timely treatment are critical for improving outcomes in sepsis patients, yet there is currently a lack of effective biomarkers in clinical practice to aid diagnosis and guide treatment decisions (3).

With the advancement of next-generation sequencing technologies, particularly the application of high-throughput sequencing, vast amounts of gene expression data have opened new avenues for identifying biomarkers of pediatric sepsis (4, 5). Significant differences in whole blood gene expression have been identified between pediatric sepsis patients and healthy controls (6, 7), enabling further screening for biomarkers capable of distinguishing between diseased and non-diseased states. These biomarkers not only facilitate early diagnosis of pediatric sepsis but may also reveal underlying molecular mechanisms of the disease, offering novel therapeutic targets (8).

Machine learning algorithms, particularly ensemble learning methods, are widely used for identifying biomarkers and constructing disease prediction models due to their ability to handle complex data and improve predictive accuracy (9, 10). These methods excel at processing high-dimensional data, managing missing values and identifying non-linear relationships, making them invaluable in biomarker research (11). However, the performance of different machine learning methods varies across different datasets (12). Therefore, employing multiple machine learning approaches for integrated analysis and selecting the optimal predictive model is a vital strategy for identifying high-quality biomarkers.

In this study, we integrated six bulk RNA-seq datasets related to pediatric sepsis. We then employed bioinformatics and 14 machine learning methods to identify sepsis biomarkers. Additionally, we examined the biological functions of these biomarkers and their connections to immune cells. Compared to previous studies, our research significantly increased the number of included datasets and the range of applied machine learning methods. We anticipate that our work will yield novel, more accurate biomarkers and predictive models for the early diagnosis and treatment of pediatric sepsis.

## 2 Materials and methods

### 2.1 Data collection and removal of batch effects

All pediatric sepsis-related bulk RNA-seq datasets and corresponding clinical information were obtained from Gene Expression Omnibus (GEO), including GSE4607 (13), GSE8121 (14), GSE9692 (15), GSE13904 (16), GSE26378 (17), and GSE26440 (17). We excluded samples with incomplete or missing clinical pathological information and ensured that the included samples were unrelated to any other diseases. Ultimately, data from 497 pediatric sepsis patients and 116 healthy whole blood samples were identified for analysis. The detailed clinical meta-information for all included samples is summarized in **Supplementary Table 1**.

We employed the Combat method from the sva package (version 3.4.6.0) (18)to remove batch effects from these six datasets using an empirical Bayesian correction model. We then performed t-distributed stochastic neighbor embedding (t-SNE) clustering analysis on the samples from each dataset using the Rtsne package (version 0.16) (19) to assess whether batch effects were successfully removed.

### 2.2 Weighted gene co-expression network analysis

WGCNA is a systems biology method for describing gene association patterns across different samples. It considers the strength and weight of relationships between nodes (genes) and uses the topological overlap matrix (TOM) to measure gene correlations. This process identifies highly co-regulated gene sets. For the integrated pediatric sepsis-related datasets, we used the WGCNA package (version 1.72-5) (20)to perform optimal soft thresholding, gene clustering, module identification, and association analysis between modules and traits. Ultimately, we selected gene modules with correlation coefficients exceeding 0.5 against pediatric sepsis for subsequent analyses.

### 2.3 Machine learning model development and evaluation

We employed an in-house Python script to randomly split the dataset samples in a 7:3 ratio, forming the training set and validation set. Multiple R packages were utilized to implement 14 classical machine learning algorithms. The glmnet package (version 4.1-7) (21) was used to implement Lasso, Elastic Network, and Ridge. We selected the tenfold cross-validation framework for feature selection and model building. When constructing the Elastic Network model, the Alpha parameter was set to 0.5. The randomForest package (version 4.7-1.1) (22) was used to implement Random Forest. The NeuralNetTools package (version 1.5.3) (23) and neuralnet package (version 1.44.2) (24) were employed to implement Neural networks. The rprop+ algorithm was selected for training, with a hidden layer configuration of 30×30. The xgboost package (version 1.7.5.1) (25) was used to implement XGBoost, with a learning rate of 0.3 per iteration and a maximum tree depth of 6 to prevent overfitting. The lightgbm package (version 3.3.5) (26) was used to implement LightGBM, with a minimum data count of 7 per leaf node and an importance score threshold of 0.01. The pls package (version 2.8-2) (27) was used to implement PLS, with an importance score threshold of 0.015. The superpc package (version 1.12) (28) was used to implement SuperPC. The e1071 package (version 1.7-13) was used to implement SVM-RFE. The adabag package (version 5.0) (29) was used to implement AdaBoost, with the number of iterations set to 30. The caTools package (version 1.18.2) was used to implement GLM. The catboost package (version 1.2) (30) was used to implement CatBoost, with the number of iterations set to 30. The rpart package (version 4.1.19) (31) was used to implement Decision Tree.

To evaluate model performance, we employed the pROC package (version 1.18.4) (32) for receiver operating characteristics (ROC) analysis. The constructed models and selected features were assessed by calculating the area under the curve (AUC) values.

### 2.4 Functional annotation and enrichment analysis

Perform Gene Ontology (GO) and Kyoto Encyclopedia of Genes and Genomes (KEGG) enrichment analyses using the clusterProfiler R package (version 4.6.2) (33). Typically, a corrected p-value below 0.05 indicates significant enrichment.

### 2.5 Immunohistochemical analysis

We used the xCell online platform to evaluate the infiltration levels of 64 cell types in 613 samples (34). We implemented two immune infiltration analysis methods, MCP-counter (35) and EPIC (36), using the immunedeconv package (version 2.1.0) (37). MCP-counter quantifies the absolute abundance of eight immune and two stromal cell types in heterogeneous tissues, while EPIC predicts the infiltration proportions of eight immune cell types. We integrated the results of these three analyses to identify cell types that were differentially expressed between pediatric sepsis and healthy samples. Hub genes were correlated with immune cells based on Spearman’s correlation coefficients.

### 2.6 Validation of biomarkers in sepsis zebrafish

In order to validate the expression levels of these core sepsis biomarkers and lay the groundwork for subsequent drug screening, we created a zebrafish sepsis model. Wild-type AB strain zebrafish at 5 days post-fertilization (dpf) were randomly selected and placed in 6-well plates at a density of 30 fish per well (experimental group). Normal and model control groups were established, with 3 mL of water in each well. The model control group (intestinal) was injected with 500 ng/fish of LPS to establish a septic zebrafish model, except for the normal control group. After one day of treatment at 28 °C, 30 zebrafish were randomly selected from each experimental group to create three biological replicates. Total RNA was extracted from the zebrafish in each group using an RNA extraction kit. The total RNA concentration and purity were then determined using a UV-visible spectrophotometer. Using 2.0 μg of total RNA from the zebrafish samples, 20 μL of cDNA was synthesized in accordance with the instructions provided with the cDNA first-strand synthesis kit. The expression of the β-actin, *rora1, rora2* and *gpr183* genes was detected by qPCR (**Supplementary Table 2**), with β-actin serving as the internal reference for gene expression and the relative RNA expression levels of the *roar1, rora2* and *gpr183* genes being calculated. The statistical results are presented as the mean ± SE. Statistical analysis was performed using SPSS software. Differences were considered statistically significant at p < 0.05.

Randomly selected transgenic MPX strain zebrafish expressing green fluorescent neutrophils at 5 dpf were used. Each well (experimental group) contained 30 treated zebrafish. Normal control and model control groups were established, with each well holding 3 mL of water. Except for the normal control group, the model control group (intestinal) received an LPS injection of 500 ng/fish to establish a sepsis zebrafish model. After 5 h incubation at 28 °C, 10 randomly selected zebrafish from each experimental group were photographed under a fluorescence microscope. Data were acquired using NIS-Elements D 3.20 advanced image processing software to analyze intestinal neutrophil counts. The validity of the sepsis model was verified based on statistical analysis of this parameter. Statistical results are presented as mean ± SE. Statistical analysis was performed using R software. Differences were considered statistically significant at *p* < 0.05.

### 2.7 Biomarker application

We have developed an open-source website for real-time biomarker applications. Built using the Django 3.0.5 web framework and the Python 3.8 programming language, the site imports data into a MySQL 5.6.49 relational database. An in-house Python script matches IDs entered via forms, and the rms package in R is used to generate the nomogram plot.

## 3 Result

### 3.1 Identification of key modules and genes associated with pediatric sepsis

**Figure 1:**
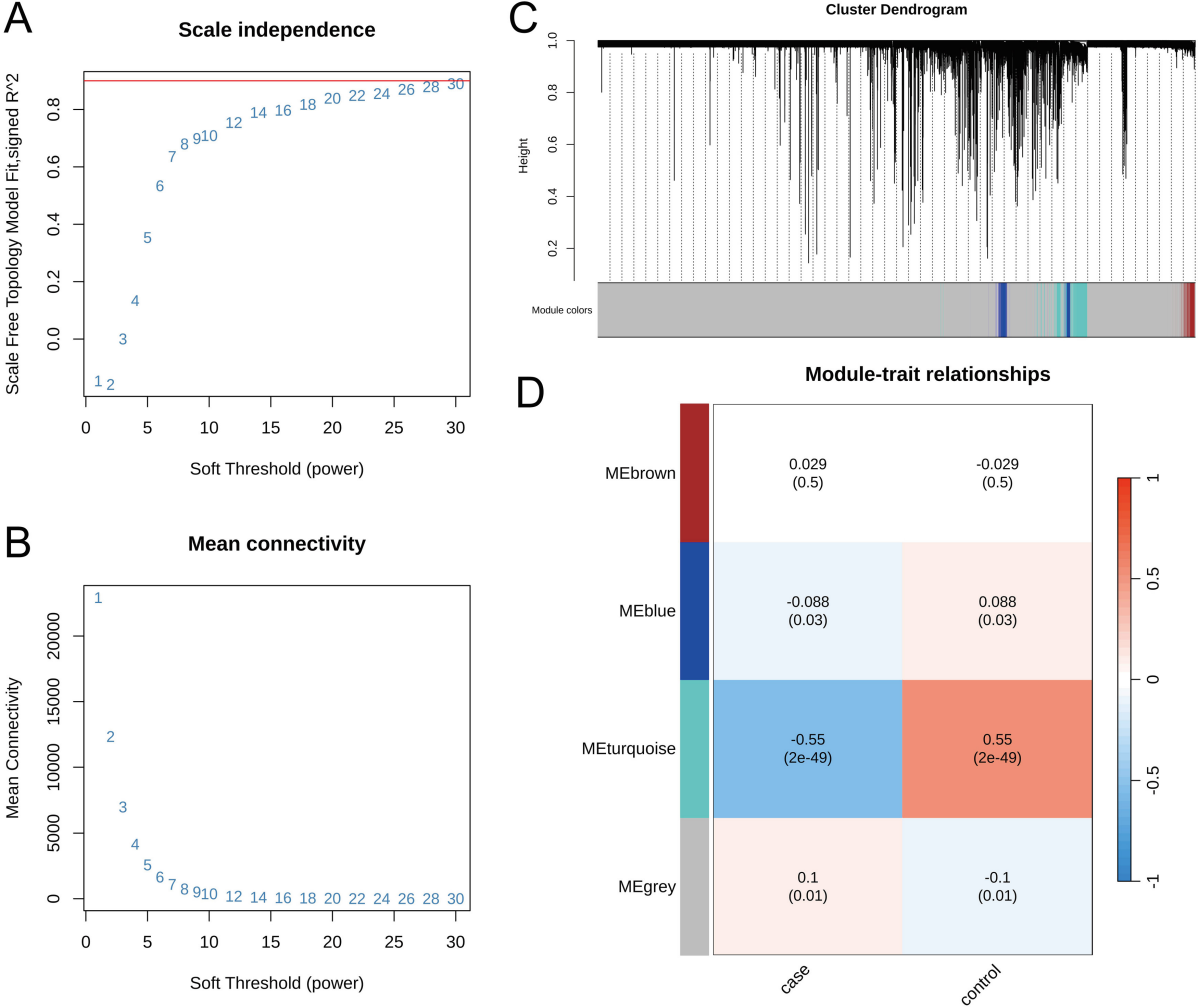
Results of WGCNA analysis. **(A, B)** Selection of the optimal soft threshold. **(C)** Construction of gene co-expression modules. **(D)** Results of the correlation analysis between gene modules and phenotypic features.

After batch effect removal using the Combat method, the integrated datasets exhibited high consistency across different GEO series, enabling robust downstream analysis (Additional **Supplementary Figure 1**). To identify modules significantly associated with pediatric sepsis, we applied WGCNA analysis to the integrated dataset. At power = 24, the signed R² reached 0.86, and the average connectivity of all nodes was at a low level, indicating that the network conformed to the scale-free network distribution (**Figures 1A, B**). Based on the optimal soft threshold (power = 24) and expression profiles, an adjacency matrix was computed to perform clustering analysis. Genes clustered onto the same branch were assigned to the same module (**Figure 1C**).

Correlation analysis between gene modules and phenotypic traits yielded the results shown in **Figure 1D**. The MEturquoise module exhibited the highest correlation with phenotypic traits, with a correlation coefficient of 0.55. This suggests that the biological functions of genes within this module may be associated with pediatric sepsis. This module comprises 1,730 genes.

### 3.2 Biomarker identification and model evaluation

**Figure 2:**
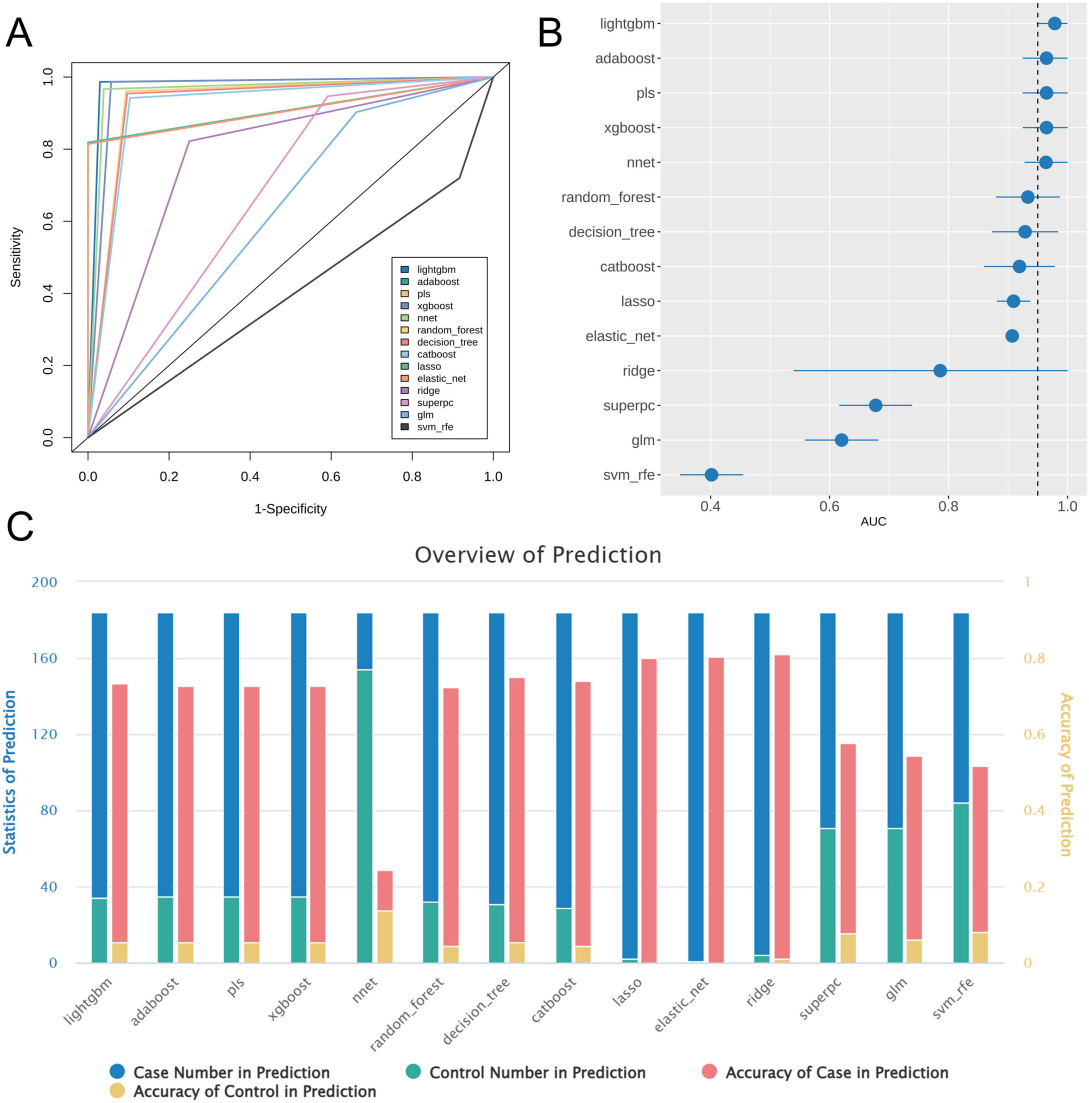
Analysis results of evaluating 14 machine learning models. **(A)** ROC curves for all models. **(B)** Forest plot of AUC values for all models. **(C)** Statistics of prediction results for the validation set across all models. The blue and green bars (left y-axis) represent the number of case and control samples predicted by each model, respectively, while the red and yellow bars (right y-axis) represent the prediction accuracy for case samples and control samples, calculated as the proportion of correctly classified samples within each group relative to the true labels. The overall accuracy of each model can be derived from the weighted sum of the red and yellow bars (red + yellow, with a maximum value of 1).

Using the pediatric sepsis-related gene modules obtained through WGCNA, we identified additional biomarkers for pediatric sepsis. We used 14 machine learning algorithms to select features, construct disease prediction models, and evaluate the performance of these models using a validation set. The importance scores of each gene derived from these optimal models are provided in **Supplementary Table 3**. ROC curves and area under the AUC values (**Figures 2A, B**) showed that models built using LightGBM, AdaBoost, PLS, XGBoost, and neural network performed best, with all AUC values exceeding 0.95. However, the neural network model had low accuracy in predicting case samples within the validation set (**Figure 2C**), likely due to the small number of control samples. Conversely, models built using LightGBM, AdaBoost, PLS, and XGBoost achieved high AUC values and demonstrated high prediction accuracy (**Figure 2C**), indicating robustness and reliability.

**Figure 3:**
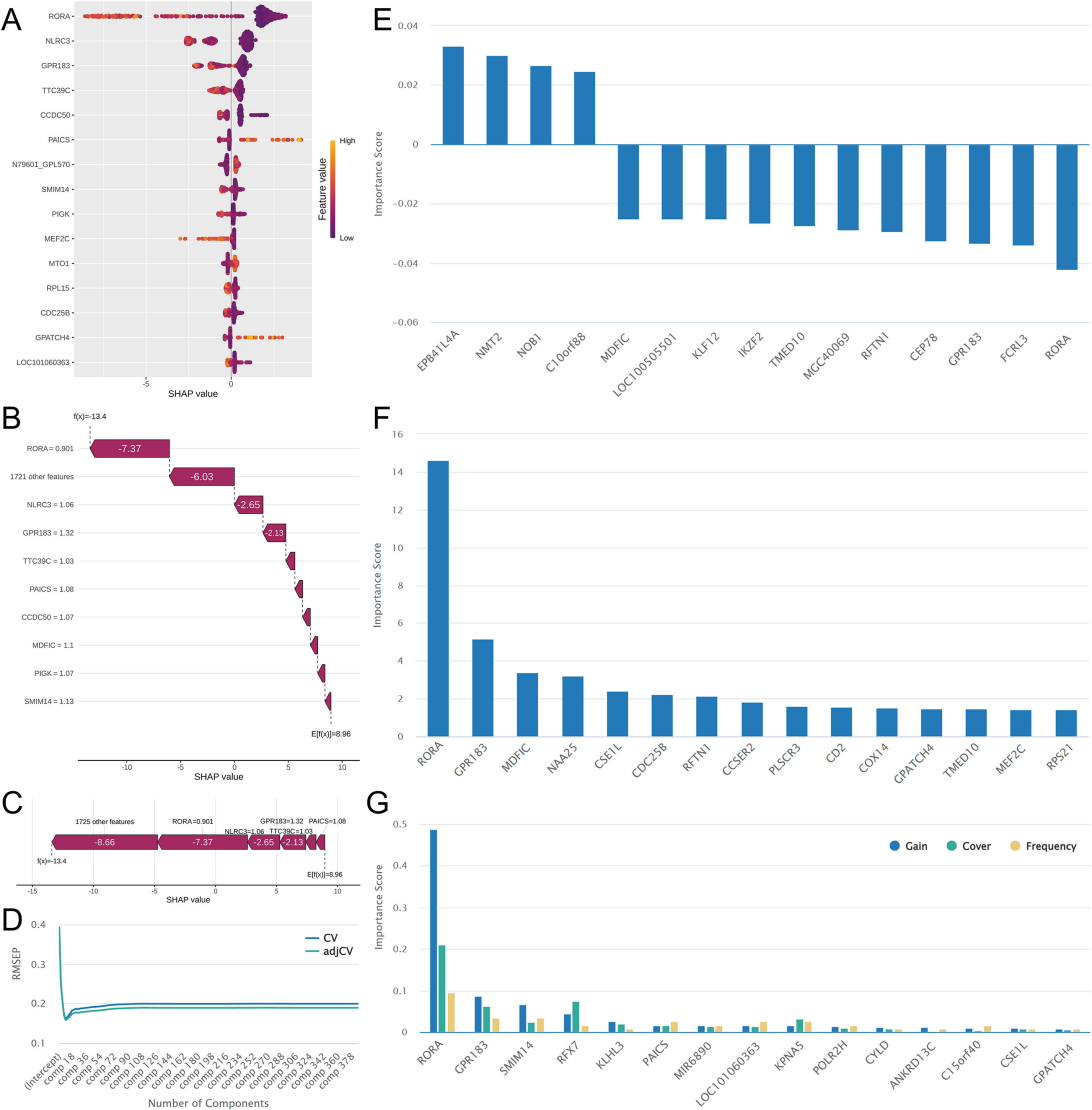
Results of the four models with optimal performance. **(A–C)** LightGBM analysis results, with SHAP interpretation demonstrating biomarker importance within the model. **(D)** RMSEP variation curve from PLS analysis results. **(E–G)** Top 15 most important genes in PLS, AdaBoost, and XGBoost models, respectively.

Of these models, the one built using LightGBM demonstrated the best performance. The Shapley Additive Explanations (SHAP) interpretation results for this model (**Figures 3A–C**) show that genes such as RORA, NLRC3, GPR183, and TTC39C have the highest SHAP values. This indicates that these genes contribute the most to the model’s predictive outcomes. Setting the number of principal components to 8 produced the lowest root mean square error of prediction (RMSEP) for the PLS model, indicating optimal performance at this setting (**Figure 3D**). Among these genes, RORA, FCRL3, EPB41L4A, and NMT2 exhibited the largest absolute coefficient values and played critical roles in the model (**Figure 3E**). In the AdaBoost-built model, genes including RORA, GPR183, MDFIC, and NAA25 achieved the highest importance scores (**Figure 3F**). In the XGBoost-built model, genes such as RORA, GPR183, SMIM14, and RFX7 had the highest values across three metrics: gain, coverage, and frequency (**Figure 3G**). These results suggest that these genes significantly contributed to improving model accuracy while demonstrating excellent data coverage and feature utilization frequency. Notably, the RORA gene achieved the highest score across all models, and the GPR183 gene consistently ranked among the top three features in all models, highlighting their importance.

**Figure 4:**
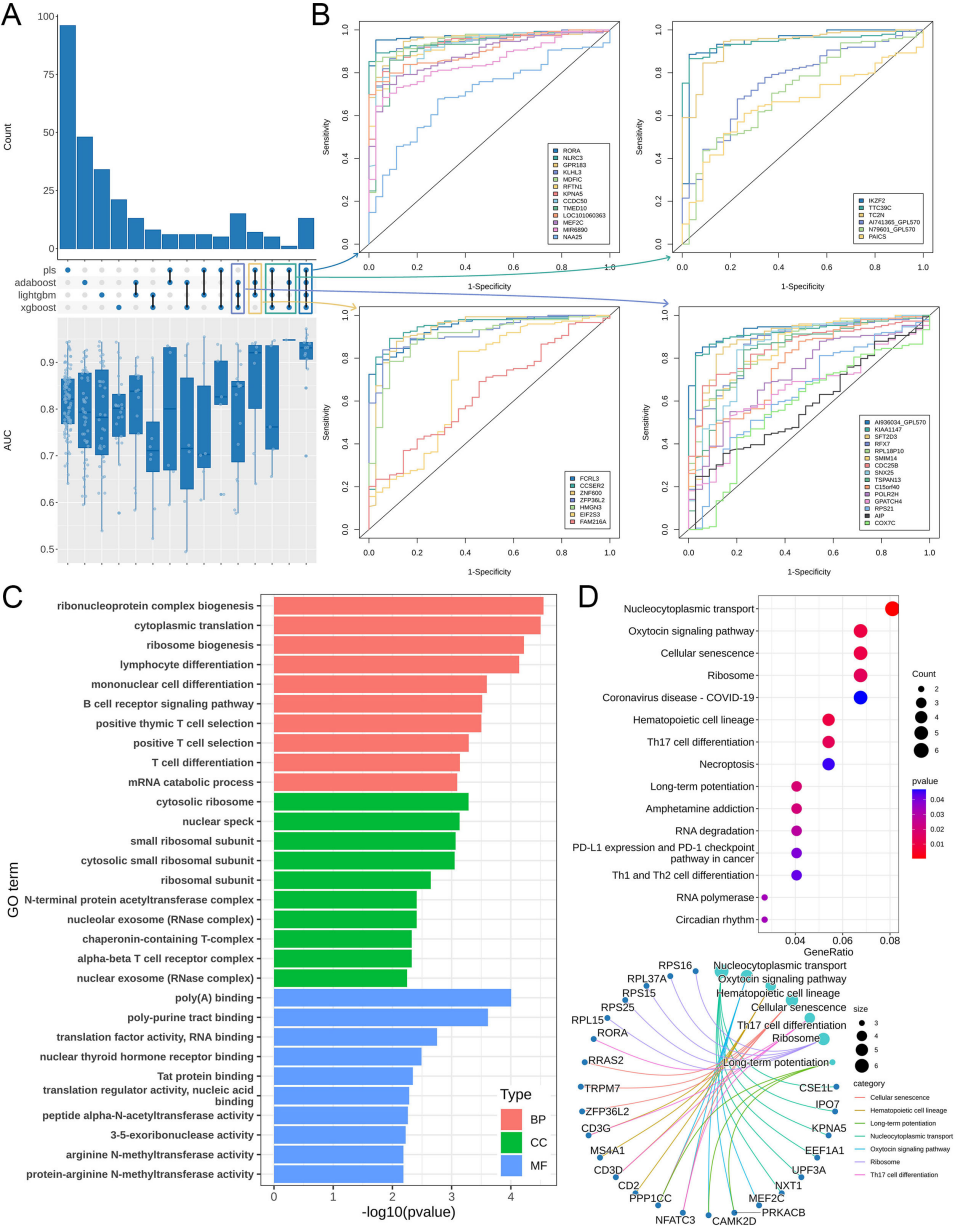
Analysis results for evaluating the diagnostic capability of biomarkers. **(A)** The upper upset plot shows the intersection of biomarkers identified by the four optimal models. The lower violin plot displays the area under the AUC values of biomarkers within each group. **(B)** ROC curves for biomarkers in the overlapping region. **(C)** GO enrichment results of these biomarkers. **(D)** KEGG enrichment results of these biomarkers.

These four models were integrated to collectively identify 284 biomarkers. We then evaluated each biomarker individually. ROC analysis revealed that most biomarkers achieved area under the AUC values above 0.7 (**Figure 4A**), particularly within the overlapping region of results from the four methods (**Figure 4B**). This indicates high predictive accuracy for these biomarkers. We then set an AUC threshold of 0.7, filtering out 237 less accurate biomarkers for subsequent analysis.

### 3.3 Functional analysis of biomarkers

To further investigate the functions of these biomarkers, we performed GO and KEGG functional annotation and enrichment analyses on the 237 selected biomarkers. Among the GO results (**Figure 4C**), the terms primarily enriched in the biological process (BP) category included ribonucleoprotein complex biogenesis, cytoplasmic translation, and ribosome biogenesis. For cellular component (CC), the highly enriched terms included cytosolic ribosome, nuclear speckle, and small ribosomal subunit. For molecular function (MF), the significantly enriched terms included poly(A) binding, poly-purine tract binding, translation factor activity, and RNA binding. KEGG enrichment analysis revealed that these biomarkers primarily participate in pathways including nucleocytoplasmic transport, the oxytocin signaling pathway, hematopoietic cell lineage, cellular senescence, ribosome, and Th17 cell differentiation (**Figure 4D**). Among these, nucleocytoplasmic transport was the most significantly enriched pathway, involving genes such as CSE1L, IPO7, and KPNA5. Notably, the RORA gene, which had the highest score across all models, also participates in the Th17 cell differentiation pathway. This suggests a strong association between Th17 cell levels and pediatric sepsis.

### 3.4 The correlation between pediatric sepsis and immune cells

**Figure 5:**
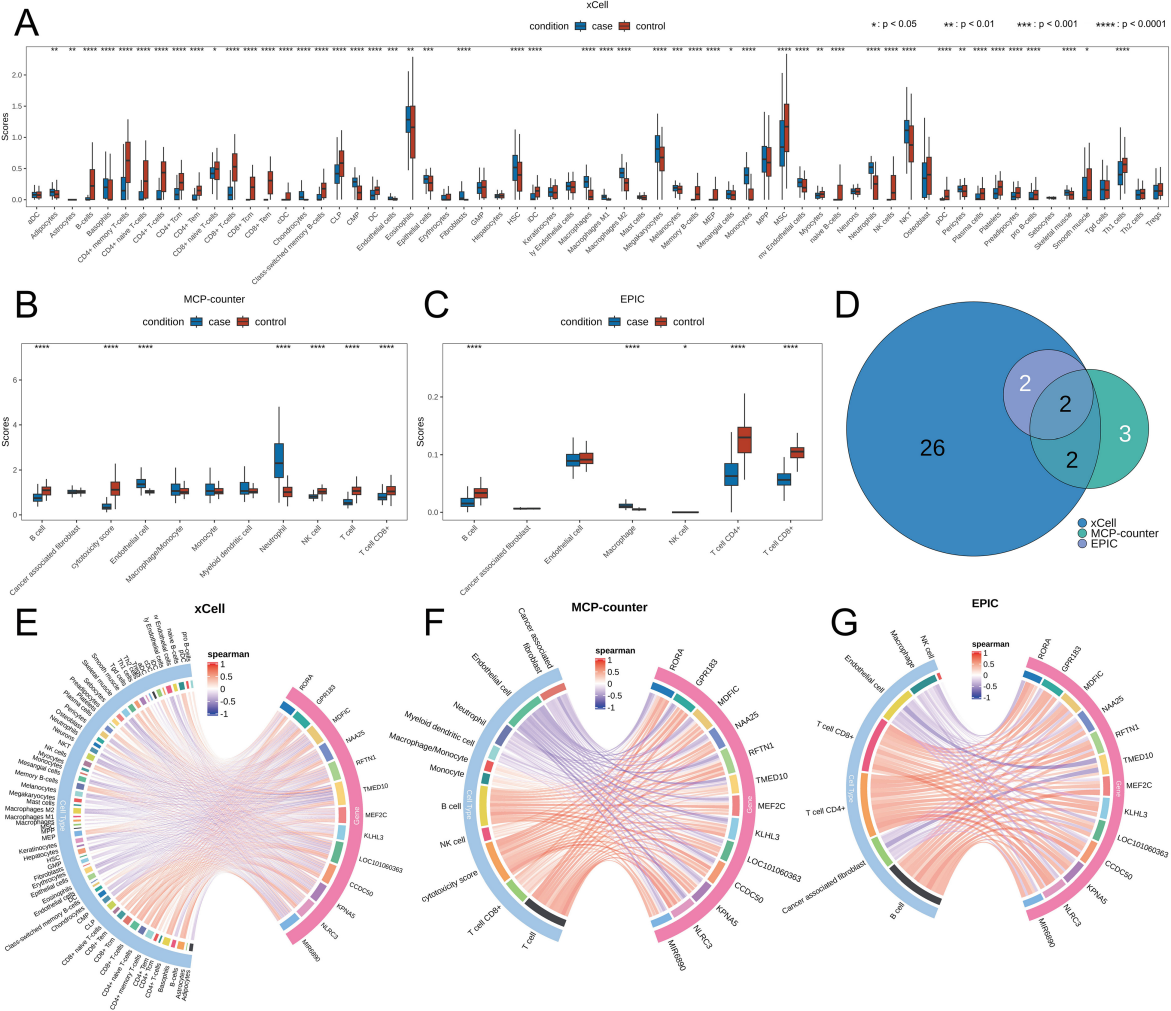
Immunological infiltration analysis results. **(A–C)** Immunological infiltration level scores for pediatric sepsis samples and healthy samples from the xCell, MCP-counter, and EPIC analyses, respectively. **(D)** Venn diagram showing cell types with significantly different infiltration levels between pediatric sepsis and healthy samples across the three analyses. **(E–G)** Results of the correlation analysis between hub genes and cell types from the xCell, MCP-counter, and EPIC analyses, respectively.

To investigate the correlation between pediatric sepsis and immune cells, we performed three types of immune cell infiltration analyses: xCell, MCP-counter, and EPIC. The xCell cell type enrichment analysis results (**Figure 5A**) showed that the infiltration levels of B cells, CD4^+^ memory T cells, CD4^+^ naive T cells, CD4^+^ T cells, CD8^+^ T cells, CD8^+^ Tcm cells, CD8^+^ Tem cells, and NK cells were significantly lower in pediatric sepsis samples than in healthy samples. Conversely, infiltration levels of HSCs, macrophages, megakaryocytes, monocytes, and neutrophils were significantly higher in pediatric sepsis samples than in healthy samples. MCP-counter results (**Figure 5B**) showed that pediatric sepsis samples exhibited significantly lower infiltration levels of B cells, NK cells, T cells, and CD8^+^ T cells than healthy samples. However, they showed significantly higher infiltration levels of endothelial cells and neutrophils. The EPIC results (**Figure 5C**) showed that B cells, CD4^+^ T cells, and CD8^+^ T cells exhibited significantly lower infiltration levels in pediatric sepsis samples than in healthy samples. Notably, both B cells and CD8^+^ T cells exhibited lower infiltration levels in pediatric sepsis samples across all three immune infiltration analyses (**Figure 5D**).

We further calculated the correlations between machine learning-screened biomarkers and various cell types. Results revealed (**Figures 5E, F**) that genes including RORA, GPR183, MDFIC, and NAA25 showed significant positive correlations with multiple immune cell types such as T cells, CD8^+^ T cells, and B cells, while exhibiting significant negative correlations with Neutrophils and Endothelial cells.

### 3.5 Validating changes in gene expression and immune cells in sepsis zebrafish

**Figure 6:**
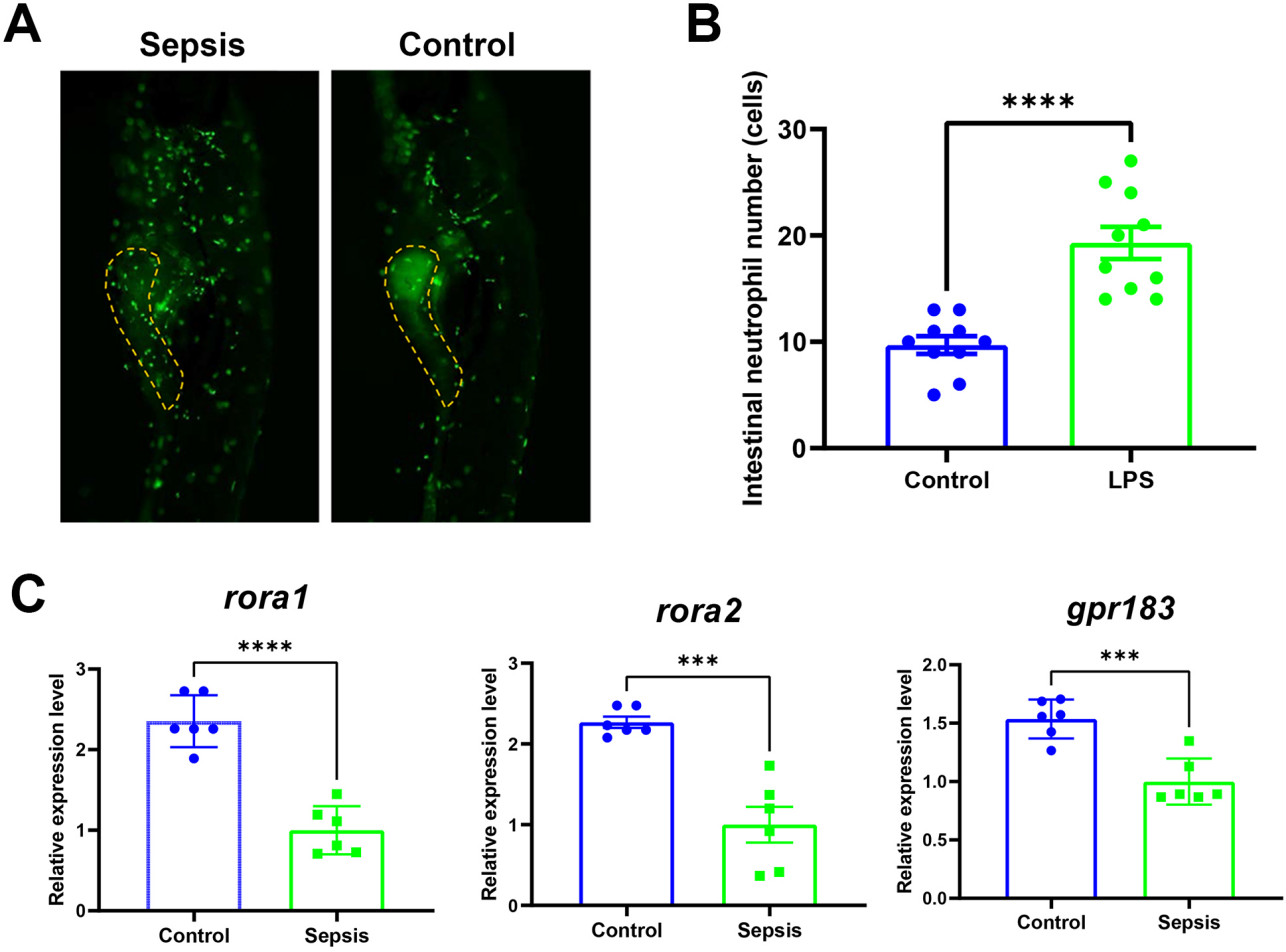
Establishment of a Sepsis Zebrafish Model and Validation of Biomarkers. **(A)** Representative images of intestinal neutrophil numbers in zebrafish from the control and sepsis groups. The yellow line-segmented area indicates the intestine, and green fluorescent dots represent neutrophils. **(B)** Statistical results of intestinal neutrophil counts in zebrafish from the control and sepsis groups. **(C)** Differences in the expression levels of *rora1*, *rora2*, and *gpr183* in zebrafish between the control and sepsis groups. *** and **** indicate significant differences at p < 0.001 and p < 0.0001.

By quantifying the number of neutrophils in zebrafish intestines, it was found that there was a significantly higher neutrophil count in the intestines of the sepsis zebrafish model compared to the control group (**Figures 6A, B**). This indicates that the sepsis model has been successfully established. Furthermore, we observed significantly downregulated expression levels of *rora1, rora2* and *gpr183* in the sepsis zebrafish (**Figure 6C**), consistent with gene expression trends observed in pediatric sepsis patients.

### 3.6 Online analysis platform for pediatric sepsis biomarkers

**Figure 7:**
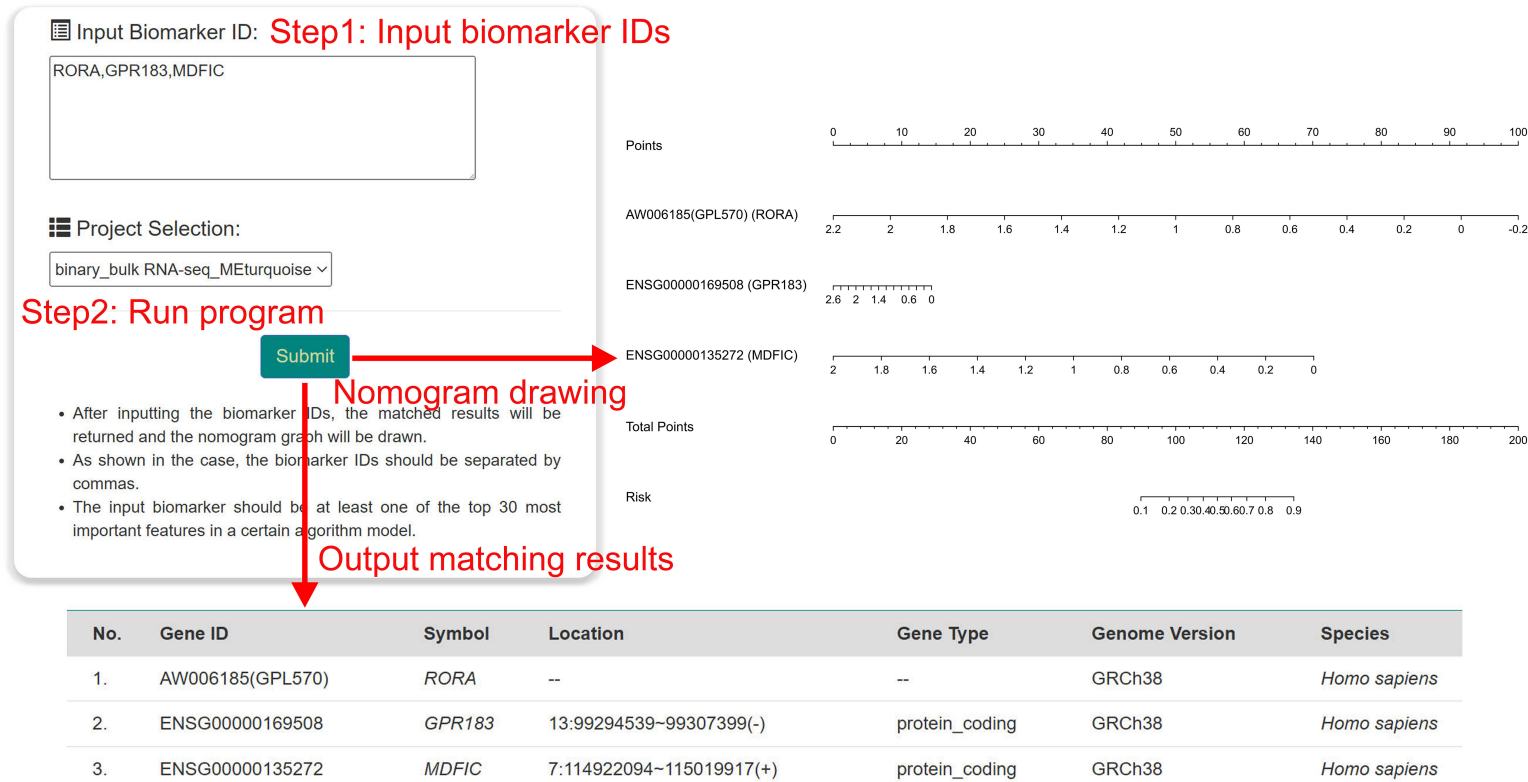
Overview of pediatric online analysis websites.

To make it more convenient for researchers to utilize our findings, we have developed an online analysis website for pediatric sepsis biomarkers (http://www.gdbioinfo.top/pdmod/nomogram/pediatric_sepsis). Users en ter the gene ID or symbol of interest on the website page and click the submit button to initiate the program (**Figure 7**). The results include a table summarizing the matched biomarker information and a bar chart based on these biomarkers.

## 4 Discussion

Pediatric sepsis, a systemic inflammatory syndrome triggered by dysregulated host immune responses, is difficult to diagnose and treat in the early stages. This study systematically identified key biomarkers associated with pediatric sepsis by integrating multi-omics data with machine learning methods and exploring their biological functions and associations with immune cell infiltration.

Using a high-quality bulk RNA-seq dataset of pediatric sepsis, we identified 237 high-confidence biomarkers. Genes such as RORA, GPR183, and MDFIC demonstrated prominent performance across multiple models. Notably, the RORA gene emerged as the highest-scoring biomarker. Its involvement in the significantly enriched Th17 cell differentiation pathway suggests it may contribute to immune dysregulation in pediatric sepsis by modulating T cell subset imbalance. This aligns with the established role of Th17/Treg dysregulation in sepsis (38, 39). Th17 cell hyperactivation has been implicated in the sepsis-associated inflammatory storm (40), and RORA, as a key transcription factor coordinating this effector cell lineage differentiation (41), may exacerbate this process by regulating IL-17 expression. GPR183 plays a key role in mediating the migration and antibody response of multiple immune cell types, including B cells, T cells, DCs, and monocytes (42). Animal studies reveal GPR183 as a critical mediator of central nervous system autoimmunity, regulating the migration of autoreactive T cells to inflamed organs (43). Thus, we suggest that GPR183 may serve as a key regulator of immune cell migration during pediatric sepsis progression, though its precise mechanism requires further functional validation.

Abnormal nucleocytoplasmic transport may exacerbate systemic inflammatory responses by affecting the nuclear export of inflammatory cytokine mRNAs (44). Recent studies have demonstrated that inhibiting the nuclear transport factor CSE1L significantly suppresses inflammation and improves acute lung injury in mice (45). Given that nucleocytoplasmic transport represents the pathway with the most significant enrichment of pediatric sepsis biomarkers, targeting inhibition of biomarkers within this pathway may improve the multi-organ inflammatory response induced by pediatric sepsis. Furthermore, these biomarkers are significantly enriched in biological processes including ribosomes, translation, the immune system, and cellular senescence, consistent with prior studies (46, 47). Transcriptomic analysis of whole blood from sepsis patients often reveals abnormal activation of ribosomal pathways, potentially linked to metabolic reprogramming in immune cells (1). Enhanced ribosomal biosynthesis may reflect the rapid proliferation demands of immune cells in early pediatric sepsis, while subsequent translational suppression may contribute to immune paralysis (48). Furthermore, the SASP has been implicated in sepsis-related organ injury (49). In summary, the biological processes implicated by these biomarkers may play crucial regulatory roles in the pathogenesis and progression of pediatric sepsis.

Immune infiltration analysis revealed significantly reduced levels of adaptive immune cell infiltration, including B cells and CD8^+^ T cells, in pediatric sepsis patients, while innate immune cell infiltration, such as neutrophils and monocytes, was increased. This phenomenon aligns with the “immune paralysis” theory of sepsis, where adaptive immune suppression coexists with excessive innate immune activation in the later stages of the disease (50). Notably, core biomarkers such as RORA, GPR183, and MDFIC showed significant positive correlations with CD8^+^ T cells, suggesting these genes may influence disease progression by regulating CD8^+^ T cell function. CD8^+^ T cell exhaustion has been demonstrated to correlate with high mortality in sepsis patients (51), and enhancing the expression levels of these core markers may potentially inhibit their apoptosis or enhance their effector functions. Furthermore, excessive neutrophil infiltration may exacerbate tissue injury by releasing neutrophil extracellular traps (NETs) (52). The negative correlation of these core biomarkers with neutrophils may provide novel molecular targets for their regulation.

This study employs a multi-dataset integration and multi-model machine learning strategy, significantly enhancing the robustness of biomarker screening. By combining WGCNA with 14 machine learning algorithms, it preserves the biological significance of gene modules while optimizing predictive performance. Furthermore, the application of the SHAP interpretation method enhances model interpretability, clarifies the contribution of key genes, and addresses the shortcomings of “black-box” algorithms (53). Notably, the model performance relies on the collective synergy of all biomarkers, rather than the individual effect of a single biomarker. This methodological framework establishes a paradigm for biomarker screening in complex diseases, demonstrating particular advantages when handling high-dimensional heterogeneous data. Notably, this study also pioneered an online analysis platform for pediatric sepsis biomarkers. However, certain limitations remain. First, the small sample size of healthy controls may introduce selection bias. Second, although batch effects were mitigated using ComBat, cross-platform data heterogeneity could still compromise result stability. Future work requires validation in large-scale independent cohorts and integration with proteomics and molecular experiments to validate transcriptomic-level discoveries.

## 5 Conclusions

This study systematically identified key biomarkers for pediatric sepsis and their potential biological functions by integrating bioinformatics and machine learning approaches, revealing their association with immune cell infiltration. An online biomarker analysis platform was also established. These findings not only deepen our understanding of the molecular mechanisms underlying sepsis but also lay a theoretical foundation for developing novel diagnostic tools and targeted therapies. Future efforts should focus on advancing these findings toward clinical translation through multidisciplinary research, ultimately improving the prognosis for children with sepsis.

## Data Availability

The original contributions presented in the study are included in the article/**Supplementary Material**. Further inquiries can be directed to the corresponding author.
